# Management of Lassa Fever: A Current Update

**DOI:** 10.7759/cureus.14797

**Published:** 2021-05-02

**Authors:** Ammar Alli, Juan Fernando Ortiz, Stephanie P Fabara, Amrapali Patel, Taras Halan

**Affiliations:** 1 Medicine, Tishreen University Faculty of Medicine, Lattakia, SYR; 2 Internal Medicine, Universitat de Barcelona, Barcelona, ESP; 3 Neurology, Universidad San Francisco de Quito, Quito, ECU; 4 Neurology, Larkin Community Hospital, Miami, USA; 5 Internal Medicine, Universidad Católica de Santiago de Guayaquil, Guayaquil, ECU; 6 Public Health, George Washington University, Washington , USA; 7 General Medicine, Ternopil National Medical University, Ternopil, UKR

**Keywords:** lassa fever

## Abstract

Lassa fever (LF) is on the top-priority infections list of both the Food and Drug Administration (FDA) and World Health Organization (WHO). This review explores the different treatment approaches found in the literature within the last 20 years. Even though ribavirin stands out among medication options, only one clinical trial was done to assess its efficacy in humans, which necessitated that we look in-depth about the latest updates in managing LF infection. For that matter, we used a Medical Subject Headings (MeSH) search on PubMed. Inclusion criteria included papers written in the English language and human subjects. Intravenous (IV) ribavirin is the most effective treatment for an acute infection. Post-exposure prophylaxis with oral ribavirin is recommended. There is not sufficient evidence to recommended convalescent plasma for the treatment of Lassa fever. LF continues to be left in the shade from global and scientific attention despite experts expecting a rise in current and future infections due to the Lassa fever virus (LFV).

## Introduction and background

Lassa fever (LF) is a life-threatening hemorrhagic infection endemic to West Africa, Sierra Leone, Guinea, Liberia, and Nigeria [1]. The etymology of the word LF is after a small village in Nigeria where the first cases were discovered in 1969 [1]. Lassa fever is a global health concern due to its significant mortality and morbidity rates [2]. However, this hemorrhagic fever's most dangerous feature is its highly contagious nature, which poses a serious risk to communities and healthcare workers alike. With the outbreak of Ebola virus disease in 2015, the world's attention to the possibility of these hemorrhagic infections transforming into global pandemics became more prominent, taken into account that they occur annually in western Africa in the form of small outbreaks [2].

Studies estimate that LF causes two million cases and 5,000 to 10,000 deaths per year [1]. However, the actual number of infections could be greater because most of the infections are mild or asymptomatic [1]. This claim is supported by the high prevalence of Lassa virus (LASV)-specific antibodies among the populations in the endemic areas [1].

LASV is a single-stranded negative ribonucleic acid (RNA) virus from the Arenaviruses (AV) family [3]. The most prominent components of LASV are its two-segmented RNA, nucleoprotein (NP), lipid envelope, and glycoprotein (GP) [2]. The virus uses a cytoskeleton-associated peptide called alpha-dystroglycan to lodge into targeted cells, usually macrophages, dendritic, and endothelial cells, where it will start its replication [2]. The virus has a silent course as it successfully inhibits the production of interferon by the infected cell through its nucleoprotein (NP). Additionally, LASV suppresses immune cells so that they do not secrete pro-inflammatory cytokines such as tumor necrosis factor (TNF)-α, IL-6, and IL-8β, contrary to what is seen in other hemorrhagic fevers [2].

Transmission mainly occurs through bodily fluid contamination of a patient infected with LASV or a reservoir animal carrying the virus [3]. The incubation period ranges from two days to three weeks [3]. LASV causes a variety of symptoms ranging from asymptomatic to mild disease in 80% of cases, which makes LASV able to continue to circulate in the community without detection [3]. The disease is usually gradual, starting with nonspecific symptoms like fever, headache, malaise, and general fatigue [3]. The condition intensifies after that, in which exudative pharyngitis, vomiting, and diarrhea are sometimes present [4]. Reports consider pharyngitis to be one of the most sensitive symptoms of LF, but its specificity is doubted [4]. Most patients develop specific antibodies early in the course of infection. However, neutralizing antibodies appear after almost weeks or months and remain low in titers [2].

While most LF patients have a mild disease, hospitalized patients have a mortality rate of 15%-20%. Complications include pleural and pericardial effusion, facial edema, neurological manifestations, and, as the name suggests, hemorrhages on mucosal surfaces and internal hemorrhages due to capillary dilatation [4].

There are no guidelines to approach patients with LF, and supportive care remains the primary method for treating it. Finding a definitive guideline or treatment will not only reduce the mortality rate but also will probably shorten the course of the infection as well. All this will help worldwide health facilities relieve the pressure on insolation units in the case of endemic or global pandemics, enabling facilities to avoid the lack of beds seen in the current COVID-19 pandemic.

This study focuses on the most promising drug ribavirin and other approaches to provide healthcare providers with the most recent updates on LF management.

## Review

Methods

For the initial gathering of information, we used the following PubMed terms: "Lassa fever/therapy" [MeSH Terms] OR ("Lassa fever"[Title/Abstract] AND "drugs" [Title/Abstract]) OR ("Lassa fever" [Title/Abstract] AND "treatment" [Title/Abstract]) OR ("Lassa fever"[Title/Abstract] AND "vaccination" [Title/Abstract]) OR ("Lassa fever" [Title/Abstract] AND "management" [Title/Abstract]).

**Figure 1:**
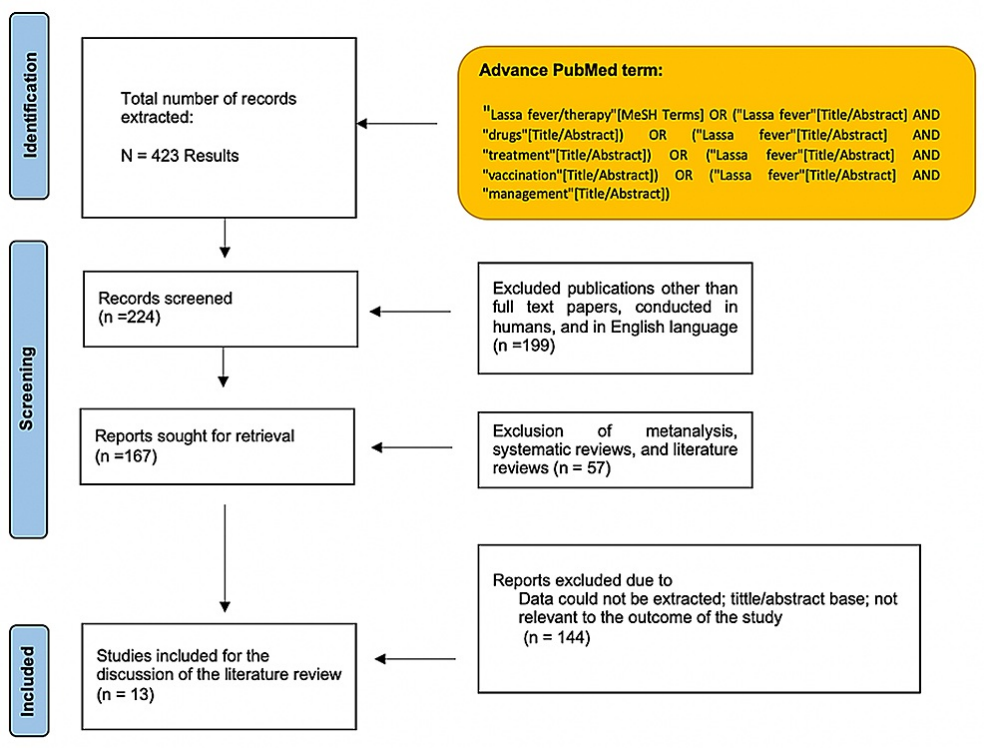
Methods and results of the study

We proceeded to do an advanced PubMed and MeSH term strategy to extract information. We used as inclusion criteria: full-text paper, conducted in humans in the English language. For exclusion criteria, we rejected literature reviews, systematic reviews, and meta-analyses. After applying the inclusion/exclusion criteria, we excluded the papers based on the title and abstract. And finally after careful analysis, we also excluded papers that did not meet our study's outcome. Figure 1 shows the step-by-step results of this literature review. 

Results

Table 1 shows the study type, intervention, and the country of the studies been analyzed in the paper [5-17].

**Table 1:** Results of the study LF, Lassa fever; EICAR, 5-ethynyl-1-b-D-ribofuranosylimidazole-4-carboxamide; MPA, mycophenolic acid; SD, standard deviation; LFCP, Lassa fever convalescent plasma; LASV, Lassa virus; CFR, coronary flow reserve.

Author, Year, Country	Study Type	Intervention	Outcome/Conclusion
McCormick et al., 1986, Sierra Leone [5]	Clinical trial	Use of IV ribavirin, oral ribavirin, and plasma	We concluded that ribavirin is effective in the treatment of Lassa fever and that it should be used at any point in the illness as well as for post-exposure prophylaxis (P = 0.0002).
Frame et al., 1984, Nigeria [6]	Clinical trial	Use of convalescent plasma	Most cases of LF who received plasma and survived showed a rapid response to therapy, in contrast with the gradual recovery in those who did not receive LFCP. Fifteen patients who received LASV convalescent plasma before the 10th day survived. Five of eight of those who received it after the 10th day died.
Ilori et al., 2019, Nigeria [7]	Cross-sectional study	Clinical features and treatment in an outbreak in Nigeria in 2018	Fatal outcomes were significantly associated with being elderly, no administration of ribavirin, and the presence of cough, hemorrhages, and unconsciousness. The p-value of the study is <0.05.
Haas et al., 2003, Germany [8]	Case report	Four cases were imported to Europa with LF. Two patients were treated with ribavirin.	The study indicates a low risk of transmission during the initial phase of symptomatic Lassa fever, even with high-risk exposures. The level of exposure was determined for 157 persons (68%), and 149 (64%) were tested serologically. High-risk or close contact was reported by 30 (19%) of 157 persons.
Ajayi et al., 2012, Nigeria [9]	Cross-sectional study	20 documented cases, 10 confirmed and 10 suspected, with LF during an outbreak in Nigeria	Patients who received ribavirin were less likely to die than those who did not (p = 0.003).
Shaffer et al., 2014, Sierra Leone [10]	Cross-sectional study	Clinical features and treatment in an outbreak in Nigeria in 2018. Comparison of early use of ribavirin vs late use of the drug	Even with ribavirin treatment, there was a high rate of fatalities underscoring the need to develop more effective and/or supplemental treatments for LF. The CFR in patients with Ag−/IgM−/IgG+ was significantly lower than that in Ag−/IgM−/IgG− patients (p = 0.045).
Buba et al., 2016, Nigeria [11]	Cross-sectional study	Clinical features and treatment in an outbreak in Nigeria in 2016	Patients who commenced ribavirin were more likely to survive (odds ratio [OR] = 0.1; 95% confidence interval [CI] = 0.03, 0.50).
Schmitz et al., 2002, Germany [12]	Case report	Two case reports of patients traveling to Africa and been treated with ribavirin	Late administration of ribavirin probably contributed to the death of both patients.
Raabe et al., 2017, Germany [13]	Case report	Two case reports of patients with LF treated with ribavirin and favipiravir	Favipiravir and ribavirin treatment of epidemiologically linked cases of Lassa fever contributed to the recovery of the two reported secondary cases.
Ölschläger et al., 2011, Germany [14]	Comparative study	In-vitro studies of EICAR and MPA	The mechanism of ribavirin, MPA, and EICAR is based on depletion of GTP, which impedes the replication of Lassa and Ebola viruses. However, this is not the predominant mechanism by which ribavirin exerts its in-vitro antiviral effect on Lassa virus.
Hulseberg et al., 2019, United States [15]	Comparative study	In-vitro studies of arbidol for LF	The study found that arbidol inhibits infection by authentic LASV, inhibits LASV GP-mediated cell-cell fusion and virus-cell fusion, and its findings suggest that arbidol inhibits LASV fusion, which may partly involve blocking conformational changes in LASV GP. The values in panel D indicate the average normalized infection in samples treated with 20 μM arbidol (±SD) from previous experiments, p < 0.01.
Zhang et al., 2020, China [16]	Experimental study	Isavuconazole, an antifungal, was used in vitro against Lassa fever virus	It was found that isavuconazole inhibits the virus at EC50 of 1.2 μM. The drug targets stable signal peptide (SSP)-membrane fusion subunit (GP2), which inhibits cell-to-cell viral fusion.
Fischer-Hoch et al., 1992 [17]	Retrospective study	Revision of side effects regarding the administration of IV ribavirin in patients infected with Lassa fever virus in Sierra Leon compared to placebo.	Ninety patients were analyzed in the study; 27% experimented rigors. Reduction of the hematocrit was seen in the second dose of treatment unrelated to the ribavirin.

Discussion

LF was first reported 50 years ago. Humanity made extensive progress in reducing mortality from the disease. However, despite modern knowledge and measures used to prevent and treat this debilitating infection, LF continues to be a threat to local communities in western Africa, where it is endemic to developing countries. New outbreaks will likely emerge if preventive measures are not carefully implemented.

Convalescent plasma 

The treatment principle is to give plasma from a previously infected patient who recovered from the infection to a patient with an active infection. Convalescent plasma has been previously successfully used in Argentine hemorrhagic fever. The disease is caused by the Junin virus, an arenavirus just like LFV [16].

A clinical trial by Frame et al. included 27 patients suspected of having LF in Nigeria. Fifteen patients received convalescent plasma 10 days before the onset of symptoms. Simultaneously, 12 patients received convalescent plasma after 10 days of the onset of symptoms [6]. All patients treated before 10 days survived, while only 75% of those treated after 10 days survived [6]. Five patients treated before 10 days were negative for the virus, and only 10 tested positive for the virus [6]. Among the patients treated after 10 days, 14 tested positive for LF, and only one patient was negative for LF [6]. Table 2 shows the main results of the clinical trial [6].

**Table 2:** Patients treated with convalescent plasma

Outcome	Treated ≤ 10 Days of the Onset of Symptoms	Treated ≥ 10 Days of the Onset of Symptoms
Died	0	8
Survived	15	4

Overall, patients who received convalescent plasma have a better outcome. Also, the patients have a more rapid recovery than the participants of the study that did not receive convalescent plasma [6]. The limitation of the study was that not all patients were confirmed to have LF at the moment of receiving the convalescent plasma.

Ribavirin and Convalescent Plasma

Ribavirin is a guanosine analog with a virus-static activity on a wide range of viruses [18]. The mechanism of action of ribavirin against LF has not been fully identified. However, studies report different ribavirin mechanisms on different viruses, including inhibition of viral RNA-dependent RNA polymerases, the inhibition of viral capping enzymes, and the inhibition of host inosine monophosphate dehydrogenase (IMDPH) [19,20].

In a study by McCormick et al., a combination of ribavirin and convalescent plasma was used in two hospitals in Sierra Leone [5]. The study evaluated the use of either oral ribavirin, IV ribavirin, or convalescent plasma.

Either medication was given if the levels of viremia were high or if the levels of aspartate transaminase (AST) were above 150 international units (IU). The AST levels were used in the study because it seems that there is a relation between the virus's levels in the blood (viremia) and AST levels. Table 3 shows the fatality rates of the study with the use of each medication [5].

**Table 3:** Fatality rates of the different treatments for Lassa fever AST: Aspartate transaminase; IU: International units; TCID, tissue culture infectious dose.

Type of Treatment	Patients Treated Within Six Days With AST Levels > 150 IU	Patients Treated After Six Days With AST Levels > 150 IU	Viremia Levels More Than 10 ^3.6^ TCID_50_
	Fatality Rate	Fatality Rate	Fatality Rate
No treatment	61%	52%	76%
IV ribavirin	5%	26%	32%
Oral ribavirin	20%	11%	30%
Plasma	38%	66%	57%

It was concluded that ribavirin was effective at any point of the illness, especially IV ribavirin. The authors also suggested that oral ribavirin should be used for prophylaxis. Convalescent plasma did not significantly reduce the mortality rate among treated patients [5].

Ribavirin

In animal studies, ribavirin showed a protective role and increased survival in infected primates, even when treatment was delayed up to five days from infection onset [5]. Ribavirin has become an accepted therapy for LF, although it was only assessed by the Centre for Disease Control and Prevention (CDC) clinical trial in 1986, hence the need for more clinical trials [19].

In 2018, an outbreak of LF occurred in Nigeria, with an estimated number of 1,893 cases, among which 423 were laboratory confirmed [7]. Ribavirin was administered to 94.1% of LF-confirmed cases [7]. The case fatality rate of patients who received ribavirin was 20.7% compared to 71.4% of those who did not receive the medication [7]. Furthermore, the study divided the patients into three groups: (1) those who received ribavirin within seven days from the onset with the lowest fatality rate, (2) patients who received ribavirin more than seven days from the onset, and (3) patients who did not receive ribavirin. The patients who received ribavirin more than seven days from the onset also had a lower fatality rate than those who did not receive ribavirin [7].

In 2000, an immigrant patient was believed to have caused secondary LF by transmitting the disease to his caring physician [8]. While examining the patient, the physician was exposed to the patient’s cough, which prompted the physician to take ribavirin for prophylaxis after the patient was confirmed to have LF [8]. The physician showed antibodies for LF. The antibodies were against LASV nucleoprotein (NP); however, because of lack of other seroconversions and because the physician did not develop symptoms, a diagnosis of secondary infection can be established [8]. However, this could be supportive of the ribavirin efficacy in protecting against LF [8].

On January 1, 2012, a case of LF presented to a hospital in Nigeria where the alert healthcare workers and the developed surveillance system resulted in a quick control of the outbreak [9]. On January 9, 2012, the outbreak was limited to 20 people, 10 confirmed and 10 suspected. Six of all the cases were nosocomial infections secondary to the presenting case [9]. The study found that the patients who received ribavirin had a lower fatality rate [9].

A study that covered LF incidence and management in Sierra Leone between 1991 and 2002 showed that LF patients who were antigen-positive, immunoglobulin M (IgM)-negative, and received ribavirin had a case fatality rate of 44% compared to 92% of those who did not [10]. It was noted that the patients who were antigen-positive, IgM-positive, and received ribavirin had better results than their counterparts treated with ribavirin [10].

A study during the 2016 outbreak, which documented the management of 47 LF patients, revealed that the patients who were given ribavirin had a lower fatality rate of 31% compared to 78.6% of those who did not take ribavirin [11].

Two case reports, the first - a 22-year-old female who traveled to western Africa with fever, flu-like symptoms, shortness of breath, and malaria - was initially diagnosed with malaria. On day nine, she was diagnosed with LF, and treatment started on day nine. However, she suffered from multiple end-organ failures due to bleeding and died on day 15 [12]. The second case - a 48-year-old male surgeon who worked for several months in a rural hospital in Sierra Leone - was also diagnosed with malaria [12]. He suffered from watery diarrhea, vomiting, arthralgia, myalgia, and crampy abdominal pain. He was diagnosed correctly with LFV on day 11 and started therapy; despite treatment, he died on day 16 due to renal failure and pulmonary infiltrates [12]. These cases suggest that ribavirin is more effective when given within six days of the start of the illness.

It is advised to give the injection in 30 minutes to prevent rigors and avoid heparin traps. Additionally, there was some report of rigors accompanied either with lumbosacral pain, headache, vomiting, or mild urticaria [17].

Favipiravir + Ribavirin

Favipiravir is a nucleoside analog designed to inhibit RNA-dependent RNA polymerase in influenza viruses [13]. The drug showed a decrease in viremia in animal studies [13]. A report on two patients who contracted secondary LF received ribavirin and favipiravir [13]. Although both patients survived, favipiravir was stopped in both patients due to nausea, vomiting, and transaminitis [13]. These adverse effects were resolved after favipiravir discontinuation and decreasing the ribavirin dose [13]. More studies on favipiravir are required to demonstrate its efficacy as a protective agent against LF.

Animal and In-Vitro Studies

Stampidine is a nucleoside derivative of d4T, a retroviral reverse transcriptase inhibitor that showed prophylactic efficacy in mice [16]. One of its peculiarities is that it penetrates the central nervous system (CNS), which may play a protective role against CNS complications of LF [16]. Further studies are needed to identify their possible efficacy in human subjects.

In a study that covered eight drugs with potential efficacy against both LASV and Ebola virus, five of these drugs (amodiaquine, arbidol, apilimod, niclosamide, and zoniporide) showed almost the same degree of inhibition of LASV and Ebola virus GP (glycoprotein) [15], while the other three (clomiphene, sertraline, and toremifene) work better on Ebola virus GP.

The study focused on arbidol since it is licensed as an anti-influenza drug outside of the United States [15]. Arbidol inhibited viral fusion with target cells through mediated GP-cell fusion and virus-cell fusion [15]. The beneficial peculiarities of these drugs are that they can be administered orally, can be stored at room temperature, show high efficacy as prophylactic agents against both dangerous viruses, and can be deployed fast and effectively during an outbreak. Some of these drugs also can be joined in cocktails that inhibit several enveloped viruses, making them particularly useful for prophylaxis before a diagnosis has been made [15].

In addition to all of the above, two new drugs that target the depletion of viral guanosine-5'-triphosphate (GTP) like 5-ethynyl-1-b-D-ribofuranosylimidazole-4-carboxamide (EICAR) and mycophenolic acid (MPA) are also effective against inosine monophosphate dehydrogenase (IMPDH) along with ribavirin; all three were effective in inhibiting the replication of LASV, but MPA and EICAR required lower concentrations to achieve that [14]. The main advantageous aspect of ribavirin compared to EICAR and MPA was that it had several mechanisms of action compared to guanosine depletion in the other two in other words; ribavirin is more resilient against possible viral resistance [14].

It was found that isavuconazole, an antifungal medication, inhibited the virus at half maximal effective concentration (EC50) of 1.2 μM. The drug targets stable signal peptide (SSP)-membrane fusion subunit (GP2), which inhibits cell-to-cell viral fusion [16].

Vaccines and prevention

The increased incidence of LF has raised the need for vaccine development. At the moment, there are 21 vaccines in the preclinical stages. Most of the vaccines are using novel technologies rather than traditional approaches [21]. In 2017 the WHO released a target profile for vaccine development, and in 2018, the Food and Drug Administration (FDA) added LF as one of their priorities for vaccine development. Among the candidates are inactive (killed) vaccines and virus-like particles such as recombinant stomatitis virus vaccine expressing glycoprotein (VSV-LASV-GPC) and LASSARAB, which is a recombinant vaccine with the rabies virus [21].

An important measure of prevention includes avoiding rodents when travelers visit endemic areas of LF and minimizing transmission from person to person [3]. The infection control of LF should be similar to other viral hemorrhagic fevers such as Ebola. Patients with LF should be isolated, and healthcare professionals should wash their hands, use contact and droplet precautions, and carefully transport body fluids [3].

Future studies and Limitations

We have found some limitations in our review; first, we only included 13 papers in the study, of which only two were clinical trials dating back to the 1980s. The number of participants in these studies was also low. Our review was mostly based on observational studies with a lesser degree of evidence than clinical trials. The reason for this was the little research done on LF in the last two decades as no clinical trials were conducted past the mentioned period. This review's main goal was to provide information about the most recent updates in LF management. Future studies should have more extensive samples. More clinical trials are needed to establish a proper treatment protocol.

## Conclusions

In conclusion, LF is an arenavirus that poses a challenging threat to the global healthcare system. Today, all studies support that IV ribavirin is the most effective treatment for acute LF, especially when administered no later than six days from the symptom onset. Oral ribavirin has proven to be efficacious as a post-exposure drug. Ribavirin was also helpful during the outbreaks, as mentioned above, in controlling the spread of infection and treating index cases. Favipiravir and stampidine were shown to have a protective role in animal studies. However, further studies need to be conducted on the drug in humans. Moreover, arbidol and drugs with similar pharmacology promise a great change in infection control and prophylaxis of various kinds of enveloped viruses including the two dangerous ones like Ebola and LASV. EICAR and MPA are only superior to ribavirin by their lower dose but not enough to make them a good substitute for it. While many papers reported favorable findings on the use of convalescent plasma, the data is still lacking enough evidence for its benefits, requiring further investigation in this regard.
